# Unusually Extensive Furuncular Myiasis in a Returning Traveller from Rural Ethiopia Complicated by Streptococcus Pyogenes Secondary Infection Following Albendazole Therapy

**DOI:** 10.3390/reports9010019

**Published:** 2026-01-08

**Authors:** Diva Jhaveri, Alastair McGregor, Matthew J. W. Kain

**Affiliations:** 1 Department of Infectious Diseases and Tropical Medicine, London North West University Healthcare, London HA1 3UJ, UK; 2 Institute of Naval Medicine, Gosport PO12 2DL, UK; 3 Department of Clinical Sciences, Liverpool School of Tropical Medicine, Liverpool L3 5QA, UK

**Keywords:** tropical medicine, furuncular myiasis, Tumbu fly, *Cordylobia anthropophaga*, Ethiopia

## Abstract

**Background and Clinical Significance**: Furuncular myiasis is a tropical parasitic skin infestation caused by dipterous fly larvae, most commonly affecting travellers to endemic regions. While returning travellers typically present with one or few lesions, extensive parasitism is rare. Increased global mobility and expanding ecological range of myiasis-causing species underscores the need for clinicians in endemic and non-endemic regions to recognise, diagnose, and manage this condition promptly. Awareness of exposure risks—including soil contact, infested clothing, and poor living conditions—is essential to reducing morbidity and preventing complications like secondary bacterial infection. **Case Presentation**: A healthy male in his forties returned to the UK after a month-long visit to rural Ethiopia, during which he slept on dirt floors and hung his washing on a line. He developed pruritic papular lesions that progressed to erythematous furuncles with central puncta and purulent discharge, accompanied by sensations of movement. The patient self-extracted 12 larvae in Ethiopia and subsequently sought local medical attention, receiving Albendazole, after which emerging larvae were non-motile. On UK presentation, he had 27 lesions at varying stages, 3 with signs of secondary infection. Laboratory investigations revealed elevated inflammatory markers, and wound swabs grew scanty *Streptococcus pyogenes.* Management included wound occlusion and systemic antibiotics. No further larvae were retrieved, precluding definitive speciation. All lesions improved over subsequent reviews. **Conclusions**: This case illustrates an unusually extensive presentation of presumed *Cordylobia* spp. myiasis in a returning traveller, highlighting potential complications following larvicidal therapy. Clinicians should maintain a high index of suspicion for myiasis in patients with compatible cutaneous lesions and relevant history. Increasing travel and shifting vector distributions make familiarity with tropical dermatoses and provision of effective safety measures essential in clinical practice.

## 1. Introduction and Clinical Significance

Myiasis is the infestation of human tissue by dipterous fly larvae. In temperate regions, the predominant aetiology of myiasis is a non-obligate species affecting wounds and necrotic tissues. In the tropics, by contrast, furuncular (subcutaneous) myiasis in travellers is predominantly caused by obligate ectoparasites. The causative species of obligate fly varies by region: *Cordylobia anthropophaga* (Tumbu fly) or *Cordylobia rodhaini* in Sub-Saharan Africa, *Dermatobia hominis* (human botfly) in Central and South America, and occasionally *Cuterebra* spp. botflies in Northern America. Other fly species such as *Chrysomya bezziana* (Old World screwworm; Asia, Africa, and the Middle East), *Cochliomyia hominivorax* (New World screwworm; Americas), and *Wohlfahrtia magnifica* (Africa) typically infest existing wounds, rather than de novo tissue invasion to complete their lifecycle.

In Sub-Saharan Africa, furuncular myiasis is most frequently caused by *C. anthropophaga*, commonly known as the Tumbu or Mango fly [1]. The true prevalence of human myiasis is unknown. Evidence is limited to retrospective case series [1,2], frequently reported from non-endemic countries in returning travellers. The impact of climate change on human myiasis is unknown, although the suitable habitat for species affecting animals has been predicted to increase [3,4].

Here, we present the case of a patient with furuncular myiasis presenting with 27 skin lesions following travel to rural Ethiopia. Two aspects of this case are unusual: First is the extensive number of lesions, with previous literature on myiasis in Africa reporting a mean of 4.3 skin lesions, with 55% being single lesions [5]. Second is secondary bacterial infection, uncommon in myiasis, which may hypothetically relate to prior oral larvicidal therapy through larval death in situ.

## 2. Case Presentation

A male in his 40s, with no known co-morbidities, presented to the emergency department of a London hospital with multiple skin lesions following recent travel to rural Ethiopia to attend a religious ceremony, including baptism in a local lake. He flew in April to Addis Ababa, before travelling through Hawassa and Yirgalem. He reached his destination, a remote village, by bus, motorcycle, and horse.

During his stay, the patient and his family members slept on bare dirt floors, walked barefoot, obtained multiple insect bites, swam in the local lake, and drank local tap water. They washed their laundry by hand and hung it on a line.

Approximately one week into his trip, the patient noted the gradual development of itchy, papular lesions on his arms, legs, feet, and lower back. Several lesions displayed a central punctum. Over subsequent days, the lesions became erythematous and started oozing purulent discharge. This was accompanied by the sensation of movement under his skin. He subsequently manually expressed 12 live larvae from various lesions. His family members experienced similar symptoms.

He sought local medical care, where he was prescribed albendazole (400 mg daily) for three days. Following completion of this treatment, he noted that further emerging larvae were non-motile. He reported a single day of fevers, and the new pustular lesions continued to develop, with some sites becoming increasingly painful and inflamed. He flew back to the UK and immediately presented to the hospital.

**Figure 1:**
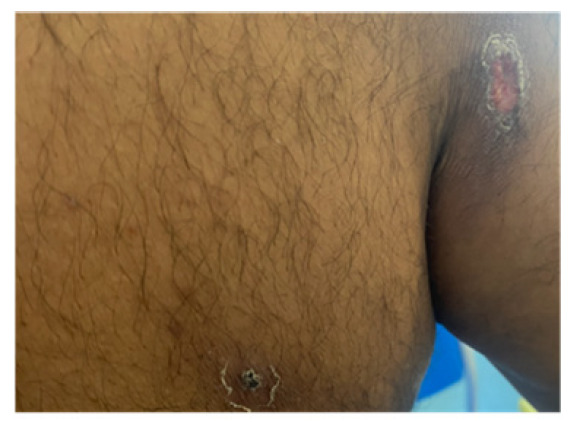
Healing lesions.

**Figure 2:**
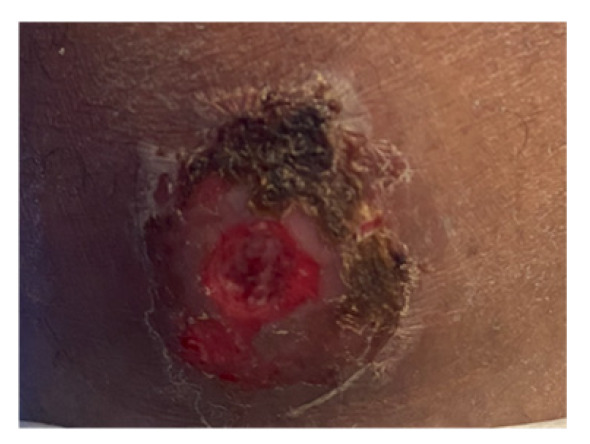
An erythematous, persistent lesion with central punctum.

**Figure 3:**
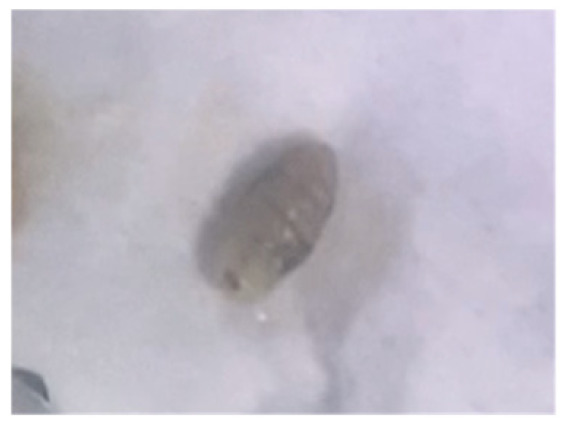
Still from patient’s video of extracted larvae.

On examination in the emergency department, the patient was systemically well and afebrile. Multiple lesions were visible on the torso, feet, and lower legs. Lesions were of various ages and stages of healing [Figure 1], with all having a central punctum, most crusted or flattened, but with some remaining open and oozing purulent material [Supplementary Appendix S1]. A few had significant surrounding localised erythema, swelling, and tenderness [Figure 2]. The patient provided videos and photos of larvae extracted in Ethiopia [Figure 3].

As no further larvae were expressed in the UK, formal speciation was not possible. Images showed cylindrical larvae with densely arranged spines across the body. This, combined with epidemiological features, led to a presumptive diagnosis of *Cordylobia* spp. myiasis.

In total, 27 skin lesions were observed across multiple body locations that were compatible with furuncular myiasis. Nineteen were reported to have already expressed larvae, and three of these were concerning for a possible secondary bacterial infection.

### 2.1. Treatment

The patient was reviewed the next day in a rapid-access infectious diseases clinic. The patient exhibited low-grade fevers, and laboratory tests showed a leukocyte count of 12.3 × 10^9^/L [normal range 4.0–11.0 × 10^9^/L] and C-Reactive Protein of 97 [normal range ≤ 4 mg/L], with all other tests within normal range. Petroleum jelly was applied to multiple non-healed lesions that were painful or where the patient had reported subcutaneous sensations. Occlusion of the lesions did not result in spontaneous expulsion of any more larvae. Additional management included wound care and dressing, analgesia, and ceftriaxone, followed by co-amoxiclav to cover secondary bacterial infection for three particularly concerning lesions. Subsequent wound swabs isolated scanty growth of *Streptococcus pyogenes* and blood cultures remained negative. The patient was followed up with improvement in his lesions, and blood tests performed 3 days later showed a completely normalised white cell count, an absence of fevers, and CRP improvement to 23 mg/L.

### 2.2. Outcome and Follow-Up

The patient was followed up for 12 more days in the rapid-access infectious diseases clinic, where all lesions were observed to be improving and healing, and he reported to be feeling well. He reported returning to work one week after his return to the UK, and remained well at further remote follow-up at 3 months, with only 2 lesions remaining, which were deemed to be flat or near flat and healing.

## 3. Discussion

We present here an unusual case of extensive myiasis in a returning traveller from Ethiopia. Definitive speciation was not possible, but the patient was given a presumptive diagnosis of *Cordylobia* spp. myiasis due to clinical features, epidemiology, and larval morphology. *C. anthropophaga* (Tumbu fly) myiasis is the most prevalent species reported from Sub-Saharan Africa [1].

The two most common causes of human myiasis worldwide, *Dermatobia hominis* and *Cordylobia* spp., typically have differing numbers of lesions. One retrospective study reported a mean number of lesions of 1.32 and 4.3, respectively [5]. A previous systematic review of human myiasis in Sub-Saharan Africa has also shown that *C. anthropophaga* cases typically cause limited disease, with most affecting a single body site [1].

There have been few reports of such extensive myiasis in the literature, especially in a travelling population. One report of myiasis by *C. anthropophaga* in women living in the Niger Delta reported extracting 98 larvae from a single patient, associated with the use of soiled linen [6] whilst one case in an Italian returning from Ethiopia over 30 years ago reported 150 larvae from *C. rodhaini* myiasis [7].

Reports of Tumbu fly myiasis in Ethiopia are limited, with most cases reporting a limited number of lesions [8,9,10,11]. As well as causing disease in humans, related species cause a significant burden on livestock in Ethiopia, with subsequent economic impact [12]. As an outbreak of New World Screwworm myiasis (causative organism *Cochliomyia hominivorax*) spreads in both humans and animals through the Americas [13], this case serves as a timely reminder of the risk to travellers and residents of endemic areas.

The lifecycle and presentation of myiasis have been reviewed extensively elsewhere [14]. Tumbu fly myiasis occurs when larvae penetrate intact skin, typically from clothing or contact with soil contaminated with eggs, both potential routes of entry for our patient. The larvae invade the dermis and develop through multiple instars, before exiting the human host to pupate.

Myiasis, as in this case, is easily diagnosed on clinical grounds, with a relevant exposure history and with specialist examination of larvae-confirming species. Ultrasound may help in the diagnosis and can help differentiate furuncular myiasis from pyogenic furuncles. Furuncular myiasis is usually self-limiting and results in full resolution of the lesion. However, complications include secondary bacterial infection, allergic reactions, pain and inflammation, as well as long-term scarring. Poor healing may also occur in immunocompromised individuals or diabetics. Lastly, this condition can also lead to psychological distress from experiencing the sensation of live organisms moving inside one’s skin.

The treatment of furuncular myiasis involves manual removal or suffocation techniques (e.g., petroleum jelly occlusion). There remains limited data on the efficacy of albendazole or ivermectin in killing larvae, and national guidelines do not recommend their use [15]. There have been no randomised clinical trials to support their use, whilst only a single prospective observational study in 25 adult patients with necrotic head and neck cancer wounds used a regimen of ivermectin, albendazole, and clindamycin for 5 days combined with wound dressing [16].

Of the 18 lesions from which larvae had been previously expressed, 9 demonstrated complete healing at review, corresponding to a 50% healing rate. In contrast, only 1 of the 9 lesions in which larvae were not expressed had healed (11%). The higher proportion of healed sites following larval expression (50% vs. 11%) may suggest that in situ larval death following albendazole administration could contribute to delayed healing or an increased risk of secondary infection; however, this association cannot be confirmed based on the available data.

In this patient, *S. pyogenes* was isolated in a wound swab from a lesion clinically concerning for secondary infection. Secondary bacterial infection is uncommon in myiasis, similar to when artificial myiasis is used as maggot therapy for debriding necrotic wounds. This has been hypothesised to be due to suppression of pathogenic bacteria by the larvae in some species but has never been proven in furuncular myiasis [17]. Hypothetically, the use of oral larvicidal medication, as with our patient, risks the larvae dying inside the furuncle, which may lead to secondary infection.

Of the 27 lesions identified in this case, 25 (93%) were located on the posterior or postero-lateral aspects of the body, including the torso, arms, and legs, as well as the patients’ feet. This distribution suggests that the primary point of exposure in this case may have prolonged contact with the floor or soil while lying down, facilitating direct infestation at these sites.

## 4. Conclusions: Learning Points/Take-Home Messages

With increasing travel to tropical endemic regions, clinicians in both endemic and non-endemic countries must maintain a high index of suspicion for myiasis in patients presenting with cutaneous furuncular lesions. Significant or prolonged exposure, as seen in this case and in previously reported cases [6,7], can lead to extensive parasitism with multiple lesions, resulting in a substantial clinical burden.

Treatment includes occlusion of the central punctum of the lesion to restrict larval oxygen and stimulate spontaneous expulsion. In some cases, surgical management may be required. There is limited evidence to support the use of oral larvicidal therapies. Secondary bacterial infection is a recognised complication of myiasis.

As such, prompt recognition, appropriate management, and traveller and physician education are essential to reduce morbidity and complications associated with parasitic skin infestations.

## Data Availability

The original contributions presented in this study are included in the article/Supplementary Material. Further inquiries can be directed to the corresponding author.

## Supplementary Materials

The following supporting information can be downloaded at https://www.mdpi.com/article/10.3390/reports9010019/s1. Supplementary Appendix 1: Summary of lesions observed at initial assessment in UK.

## Abbreviations

The following abbreviations are used in this manuscript:

CRP	C-Reactive Protein
