# Supplementary material for: Unusually Extensive Furuncular Myiasis in a Returning Traveller from Rural Ethiopia Complicated by Streptococcus Pyogenes Secondary Infection Following Albendazole Therapy
Source: Reports (MDPI). 2026 Jan 8;9(1):19. doi: 10.3390/reports9010019 (PMC12821619; doi:10.3390/reports9010019)
Supplement: Supplementary file 1 [file reports-09-00019-s001.zip › reports-4087138-supplementary.pdf]

Supplementary Appendix 1: Summary of lesions observed at initial assessment in UK

Location on body	Larvae expressed prior to assessment	Status at initial assessment in UK
Left foot - dorsum	No	Shallow ulcer not healed
Left ankle - lateral maleolus	No	Shallow ulcer not healed
Right ankle - anterior	No	Shallow ulcer not healed
Right calf - posterior lateral (inferior lesion)	Yes	Significant surrounding erythema 10x10 and swelling – possible secondary infection
Right calf - posterior lateral superior	No	Shallow ulcer not healed
Right leg - lateral distal femur	Yes - twice	Some surrounding erythema and swelling
Left upper outer gluteal region - multiple lesions	Yes - one each	Healed
Right upper outer gluteal region	Yes	healed
Left posterior chest - scapula	Yes	Healed
Right posterior chest - scapula superior	Yes	healed
Right posterior chest - scapula inferior	Yes	Healed
Abdomen - Right lower quadrant	Yes	Healed
Right tricep	Yes - twice	Open but healing shallow ulcer
Right deltoid - superior	Yes	Healed
Right deltoid - inferior	Yes	Healed
Right distal humerus above elbow	Yes	Ongoing ooze
Right elbow - lateral posterior	No	Ooze but improving
Right elbow - medial	No	Dry and improving
Left posterior arm - tricep superior lesion	Yes	Healed
Left posterior arm - tricep inferior lesions (multiple)	Yes	Ulcer but healing
Left elbow - posterior	No	Oozing and tender
Left elbow - lateral	Yes	Oozing and tender
Left elbow - medial	Yes	Improving
Left forearm - medial upper lesion	No	Open shallow ulcer
Left forearm - medial lower lesion	No	Open and oozing
